# Single-Molecule and Super-Resolution Diffusion Quantification Unveils Reversible Enhancement of Lipid-Membrane Diffusivity by General Anesthetics

**DOI:** 10.1021/acsnano.5c13446

**Published:** 2025-11-13

**Authors:** Tyler Jepson, Hansen Jin, Chun Ying Wu, Wan Li, Ke Xu

**Affiliations:** † Department of Chemistry, 1438 University of California, Berkeley, California 94720, United States; ‡ California Institute for Quantitative Biosciences, University of California, Berkeley, California 94720, United States

**Keywords:** single-molecule microscopy, general anesthetics, lipid bilayer diffusion, lipid bilayer order, chloride permeability, super-resolution microscopy, live-cell microscopy

## Abstract

The molecular mechanism of general anesthesia remains a mystery. While many small molecules, ranging from xenon to diethyl ether, act as general anesthetics, few similarities exist in their chemical structures or properties. Utilizing single-molecule displacement/diffusivity mapping (SM*d*M), a diffusion-quantifying single-molecule and super-resolution microscopy tool, we unveil that at clinical concentrations, general anesthetics rapidly and reversibly enhance the lateral diffusivity of both model lipid bilayers and live-cell plasma membranes in a dose-dependent fashion based on the anesthetic potency. With *in situ* fluorescence microscopy, we next show that the partitioning of anesthetics into the lipid bilayer causes fast dilation and area expansion. Employing a liposome-based fluorescence quenching assay, we further unveil enhanced lipid-bilayer permeability to the chloride ion (Cl^–^) in an anesthetic-concentration-dependent fashion. Together, our results indicate that the reversible insertion of anesthetic molecules into the lipid bilayer enhances the diffusivity and permeability of the lipid membrane, thus compromising neural functions.

## Graphical abstract

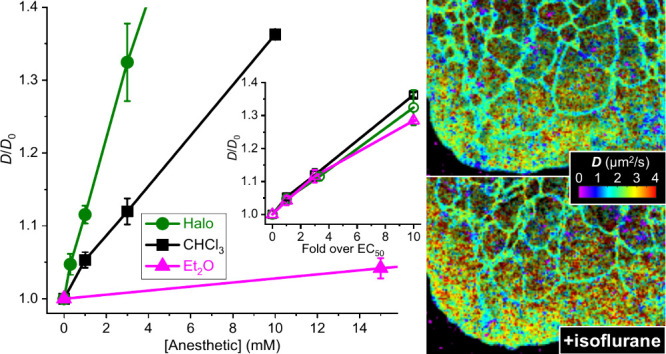

Despite ubiquitous clinical use, the molecular mechanism of general anesthesia remains a mystery.^1–7^ While many small molecules, ranging from xenon and nitrous oxide to chloroform and diethyl ether, act as general anesthetics, few similarities exist in their chemical structures or properties, except that many are sparingly soluble in water.

The strong partitioning of general anesthetics into oily phases led to early speculation that they act on the cell membrane. Notably, for diverse anesthetics, a good correlation is found between their potencies and partition coefficients in lipid bilayers over 4 orders of magnitude.^8,9^ Consequently, the half-maximal effective concentrations (EC_50_) of different anesthetics convert to similar partitioned concentrations of ∼30 mM in lipid bilayers or ∼3% mole fractions. While various studies have examined the effects of this substantial mixing on lipid-bilayer properties, generally showing reduced lipid packing order, increased fluidity, and depressed melting and critical temperatures,^10–16^ experiments are mainly on vesicle solutions and readouts are often indirect. It remains difficult to elucidate how these effects manifest themselves in cells and connect to anesthesia. Cellular membranes are characterized by substantial heterogeneities at the nanoscale, readily obscuring the likely subtle changes in membrane properties induced by anesthetics.

The discovery that luciferase activity is inhibited by anesthetics^17^ has given rise to alternative anesthesia mechanisms based on action on proteins, especially ion channels. However, despite decades of research, no unifying targets have been identified.^1–7^ Although reported stereoselectivity^18,19^ suggests protein interactions, conflicting studies have found minimal potency differences between enantiomers of volatile anesthetics.^20,21^ Above all, given the promiscuous nature of anesthetic–protein interactions, it remains difficult to rationalize why anesthesia precedes other consequences with sharp transitions at EC_50_, and why anesthetics of diverse structures and putative protein interactions produce simply additive effects when applied together.^5^

A recent study^22^ rekindled discussion on the contribution of membrane interactions to anesthesia. Through single-molecule localization microscopy (SMLM) of fixed cells, an interesting model was proposed in which anesthetics disrupt lipid rafts (liquid-ordered nanodomains) in the plasma membrane. However, the existence of lipid rafts is disputed.^23–25^ Our spectrally resolved SMLM results suggested that raft-like structures are not native to the cell membrane but may be artificially induced by labeling.^26^

Utilizing single-molecule displacement/diffusivity mapping (SM*d*M), a diffusion-quantifying super-resolution microscopy tool we recently developed,^27–29^ here we unveil that at clinical concentrations, general anesthetics rapidly and reversibly enhance the lateral diffusivity of both supported lipid bilayers (SLBs) and live-cell plasma membranes in a dose-dependent fashion based on the anesthetic potency. With *in situ* fluorescence microscopy, we next show that the anesthetic insertion dilates and dilutes the lipid bilayer, causing area expansion and extrusion. Using a liposome-based fluorescence quenching assay, we further detect enhanced permeability of the lipid bilayer to chloride ions in an anesthetic-concentration-dependent fashion. Together, our results suggest that the reversible insertion of anesthetic molecules into the bilayer loosens lipid packing, thereby enhancing the diffusivity and permeability of the lipid membrane and compromising neural functions.

## Results and Discussion

### Anesthetics Reversibly Increase the Lateral Diffusivity of Supported Lipid Bilayers

To probe the lateral diffusivity of SLBs, a minuscule amount (∼0.0003%) of fluorescently labeled lipid (Texas Red-1,2-dihexadecanoyl-*sn*-glycero-3-phosphoethanolamine; Texas Red-DHPE) was added to 1,2-dioleoyl-*sn*-glycero-3-phosphocholine (DOPC) in chloroform, with which SLBs were formed on precleaned glass coverslips.^30,31^ Glass-supported SLBs have been commonly used to examine bilayer diffusivity,^32,33^ even as glass interactions may impede diffusion. Whereas polymer-cushioned bilayers remove substrate interactions,^32^ for our experiments they are potentially complicated by anesthetic-polymer interactions. Suspended bilayers provide another means to remove substrate interactions,^34–36^ but are complicated by membrane tension, especially considering bilayer expansion upon anesthetic addition (below).

For SM*d*M,^27–29^ the sample was illuminated under total internal reflection for wide-field single-molecule imaging. An EM-CCD camera recorded at 109.3 frames per second (fps), and the excitation laser illuminated at the center of every frame as 2.5 ms duration pulses. The low illumination duty cycle allowed fresh fluorescent probes to diffuse into the imaging area from outside the illuminated region, thus maintaining a suitable density of single molecules in the field of view.^29^ The recording of ∼10^4^ frames in a few minutes allowed 10^5^–10^6^ single-molecule displacements *d* to be extracted between consecutive frames at a fixed time separation of Δ*t* = 9.15 ms. Fitting these displacements to a normal diffusion model yielded diffusion coefficient *D* with ∼±0.3% brackets for the 95% confidence interval (CI).^29,37^ For the as-prepared DOPC SLBs, SM*d*M measured *D* values of 3.5–4 μm^2^/s in phosphate-buffered saline (PBS) (Figure 1A) at room temperature (22 ± 1 °C), as expected.^38^

**Figure 1:**
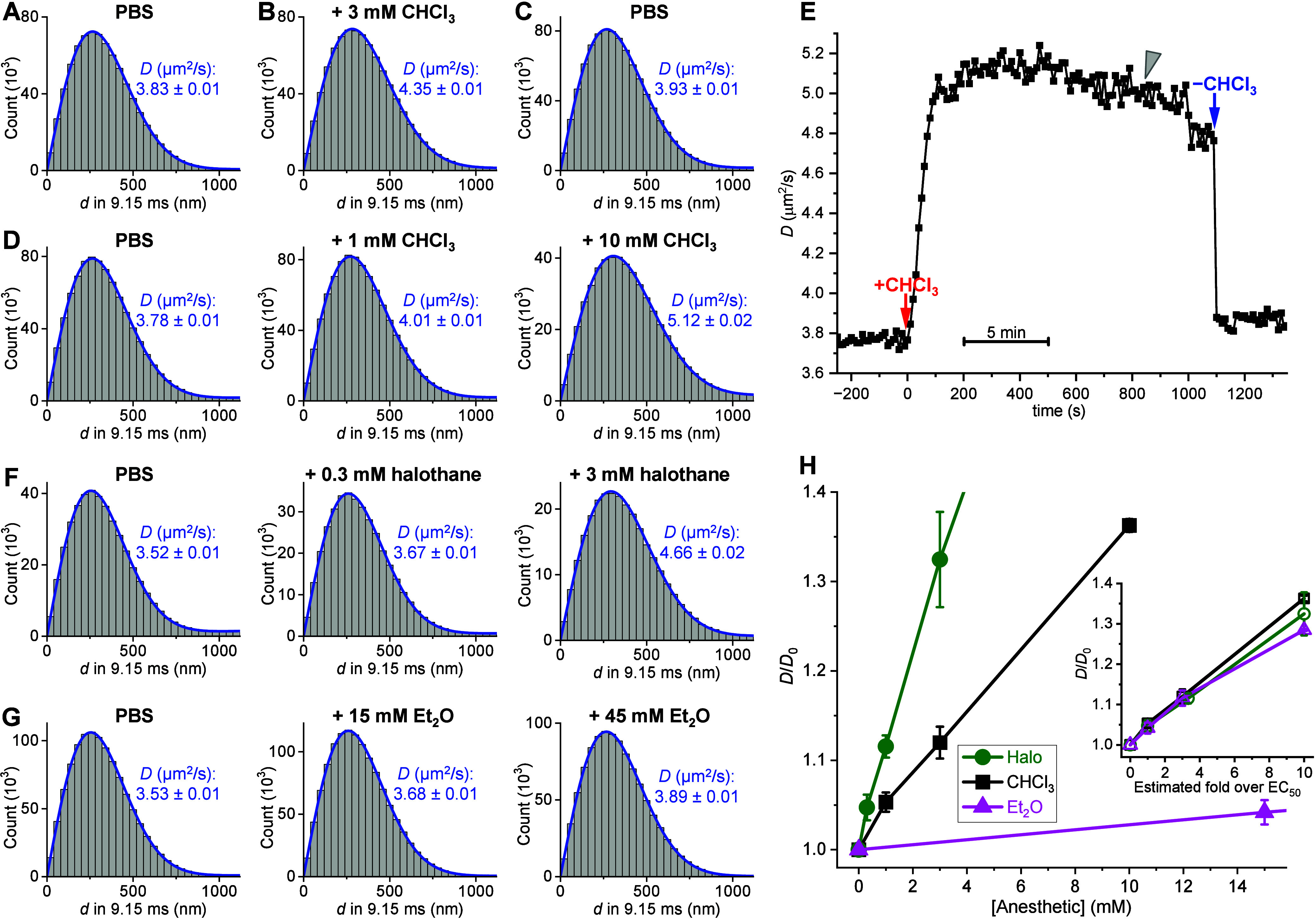
Large statistics of single-molecule displacements through SM*d*M unveil anesthetic-induced reversible increases in the lateral diffusivity of SLBs. (A) Histogram: SM*d*M-recorded distribution of 8.0 × 10^5^ single-molecule displacements *d* for Texas Red-DHPE in a glass-supported DOPC SLB in PBS, at a fixed time separation of Δ*t* = 9.15 ms. Blue curve: Fit to a normal diffusion model, yielding *D* = 3.83 ± 0.01 μm^2^/s (95% CI). (B,C) Histograms: distributions of single-molecule displacements for the same SLB after adding 3 mM chloroform (CHCl_3_) to the top PBS medium (B), and then after washing off with fresh PBS (C). Blue curves: Fits to a normal diffusion model, with resultant *D* and 95% CI marked in the graphs. (D) Similar to (A–C), but for another SLB in PBS (left), after adding 1 mM chloroform to the PBS medium (middle), and after further adding chloroform to 10 mM (right). (E) SM*d*M-determined *D* values versus time for an SLB in PBS, to which 10 mM chloroform is added at time 0 (red arrow) and removed at 1100 s (blue arrow). Each data point is from fitting to 10 s of SM*d*M data. (F) Similar to (D), but for another SLB in PBS (left) and after adding halothane to the PBS medium to 0.3 mM (middle) and 3 mM (right). (G) Similar to (D), but for another SLB in PBS (left) and after adding diethyl ether (Et_2_O) to the PBS medium to 15 mM (middle) and 45 mM (right). (H) Summary of SM*d*M-determined SLB *D* values in PBS with varied amounts of chloroform (black), halothane (green), and diethyl ether (magenta), relative to the starting values in PBS (*D*_0_). Error bars: standard deviations between 3 runs at each concentration. Inset: The same *D*/*D*_0_ values plotted against anesthetic concentrations divided off by their respective EC_50_ values, taken as 1, 0.3, and 15 mM for chloroform, halothane, and diethyl ether.

We start by examining the effects of chloroform (CHCl_3_), a classic volatile anesthetic with an aqueous-phase EC_50_ equivalence of ∼1 mM.^1,39,40^ We added chloroform (and other anesthetics below) to the top PBS medium at stated concentrations and immediately sealed the sample for SM*d*M. However, given the anesthetics’ high volatility, the actual concentrations were expectedly lower. We thus consider nominal chloroform concentrations of ∼1–3 mM to be reasonably within clinical levels.

Notably, adding 3 mM chloroform to the top PBS medium induced a 13% increase in the SM*d*M-determined SLB diffusivity (Figure 1B). This chloroform-induced *D* increase was reversible after the sample was washed off with fresh PBS (Figure 1C), and was further dose-dependent, with 1 and 10 mM chloroform enhancing *D* by 5% and 35%, respectively (Figure 1D,H). Fluorescence-spectrometer measurement of Texas Red-DHPE in PBS-suspended small unilamellar vesicles detected unvaried fluorescence emission (Figure S1) for chloroform additions up to 33 mM, suggesting no substantial changes in dye photophysics in the process.

To monitor the chloroform action kinetics, we continuously ran SM*d*M *in situ* for an SLB as we added and removed chloroform in the top PBS medium. The recorded data were temporally divided into 10 s segments for individual *D* fitting. Plotting the resultant *D* values versus time (Figure 1E) showed a rapid increase upon chloroform addition (red arrow), which plateaued in ∼1 min, followed by a gradual, minor drop over ∼10 min (gray arrowhead) attributable to chloroform evaporation. In the opposite process, replacing the top medium with fresh PBS (blue arrow) resulted in prompt *D* recovery within a single 10 s time point. The observed fast kinetics and good reversibility may be rationalized by the likely rapid molecular exchange between the solution and the ∼4 nm-thick lipid bilayer and are consistent with the known fast action and good reversibility of general anesthesia.

We next compared several other representative general anesthetics. Halothane and isoflurane have similar aqueous-phase EC_50_ equivalences of ∼0.3 mM.^1,39,40^ We accordingly observed more potent SLB diffusivity enhancements over chloroform, with 0.3 and 3 mM halothane, respectively, raising *D* by 4% and 32% (Figure 1F,H), and 0.6 mM isoflurane raising *D* by 7% (Figure S2). Meanwhile, diethyl ether is substantially less potent with an aqueous-phase EC_50_ equivalence of ∼15 mM.^39,40^ We observed ∼4% and ∼10% SLB *D* increases at 15 and 45 mM diethyl ether additions, respectively (Figure 1G,H). Notably, scaling the wide-ranging concentrations of the different anesthetics (Figure 1H) by their estimated EC_50_ values yielded similar trends in SLB *D* enhancement (Figure 1H, inset).

In comparison, 1,2-dichlorohexafluorocyclobutane (F6) is a “nonimmobilizer” that does not induce anesthesia and dissolves poorly in water.^41,42^ Dimethyl sulfoxide (DMSO) is a nonanesthetic solvent that preferably partitions in water over oil. SM*d*M showed no substantial changes in SLB diffusivity with F6 and DMSO applied at high concentrations (Figure S2).

We further examined SLBs formed with a saturated/unsaturated lipid mixture of DOPC, brain sphingomyelin, and cholesterol at a 1:1:1 mol ratio. Notably, while SM*d*M determined substantially lower *D* of ∼1.0 μm^2^/s for the mixed-lipid SLBs, as expected,^43^ chloroform application induced a similar scaling of *D*, showing ∼5% and ∼30% increases for 1 mM and 10 mM chloroform additions, respectively (Figure S3). Together, SM*d*M unveiled that general anesthetics consistently enhance the lateral diffusion of lipid bilayers in a concentration-dependent fashion related to their EC_50_ values.

### Anesthetics Reversibly Increase the Lateral Diffusivity of Plasma Membranes of Cultured Fibroblasts and Neurons

We next investigated whether the above anesthetic-enhanced bilayer diffusion manifests itself in cells. To this end, we cultured a common fibroblast cell line, COS-7, on glass coverslips, and added to the cell medium BDP-TMR-alkyne, a lipophilic dye that switches on fluorescence in the lipid phase to enable SM*d*M of cellular membranes.^28^ Rendering the localized single molecules in the SM*d*M data into SMLM super-resolution images well resolved the plasma membrane and intracellular organelle membranes at the nanoscale (Figure 2A). The SM*d*M-acquired single-molecule displacements were spatially binned onto a 120 nm × 120 nm grid. Displacements that fell into each bin were separately fit to a normal diffusion model to obtain a local *D* value, with which super-resolution *D* maps were constructed (Figure 2B).^27–29^

**Figure 2:**
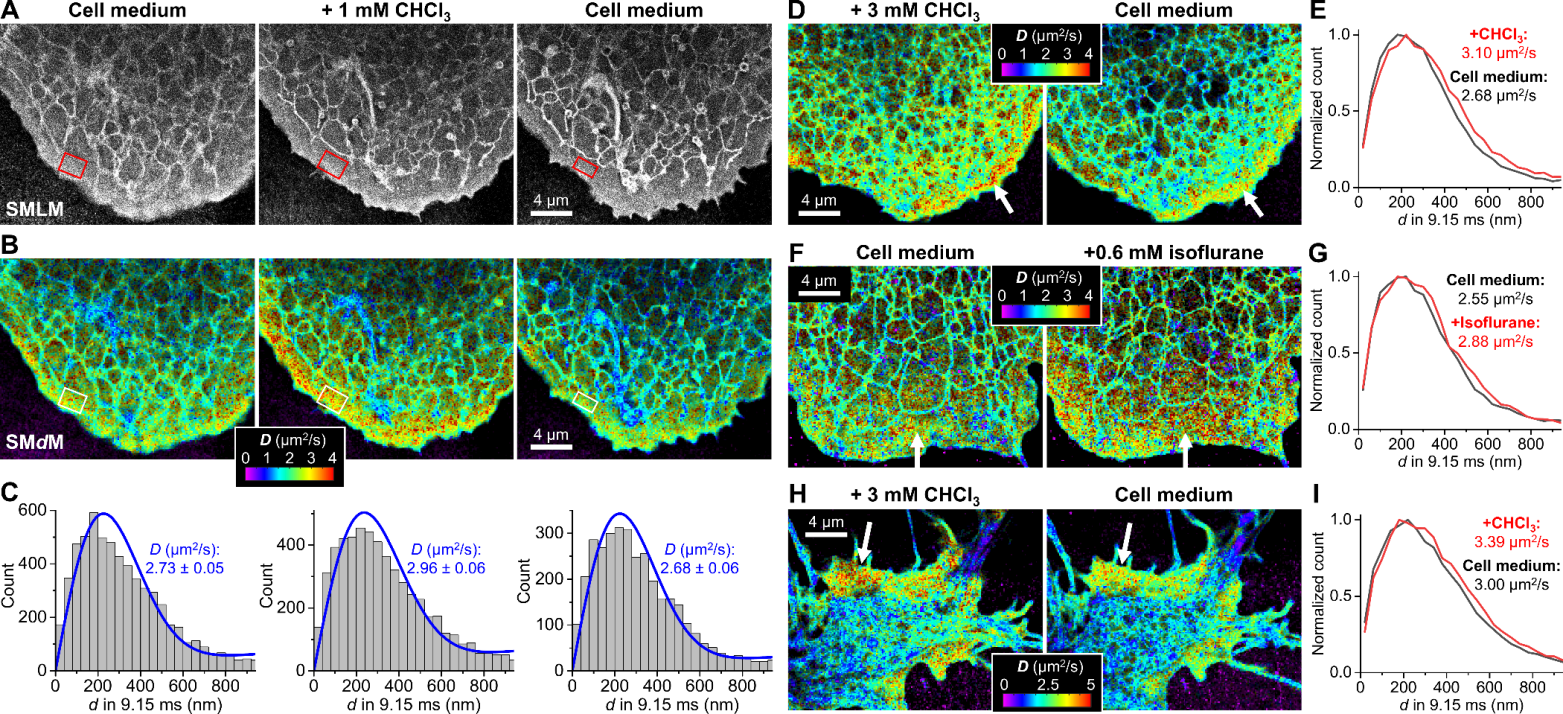
SM*d*M super-resolution diffusivity mapping unveils anesthetic-induced diffusivity increases in live-cell plasma membranes. (A,B) SMLM images (A) and color-coded SM*d*M diffusivity maps (B) of BDP-TMR-alkyne in COS-7 cellular membranes before (left) and after (middle) adding 1 mM chloroform to the cell medium, and after then washing off chloroform with medium (right). The SMLM images in (A) were constructed from the localized BDP–TMR–alkyne molecules in the SM*d*M data, whereas the diffusivity maps in (B) were generated by spatially binning the SM*d*M-acquired single-molecule displacements with a 120 nm × 120 nm grid and then fitting the displacements in each bin for a *D* value. (C) Histograms: distributions of single-molecule displacements *d* (time separation Δ*t* = 9.15 ms) for the boxed regions in (A,B). Blue curves: Fits to a normal diffusion model, with fitted *D* and 95% CI marked. (D) Color-coded membrane diffusivity maps of a COS-7 cell in the presence of 3 mM chloroform (left) and after washing off with medium (right). (E) Normalized distributions of single-molecule displacements for plasma-membrane regions of the two diffusivity maps, as pointed to by arrows in (D), to which *D* values are fitted and marked in the graph. (F,G) Similar to (D,E) but for another cell before and after adding 0.6 mM isoflurane to the cell medium. (H,I) Similar to (D,E) but for the soma of a cultured neuron.

For the resolved cell plasma membrane, SM*d*M revealed ∼8% and ∼16% increases in *D* when 1 mM and 3 mM chloroform were added to the cell medium, respectively (Figure 2B–E and Figure S4). Although cell-membrane heterogeneity and dynamics challenged quantification, the color-coded *D* maps generally exhibited upshifts in *D* with chloroform addition. Continuous SM*d*M recording further visualized fast *D* increases for the plasma membrane upon chloroform addition (Figure S5). Washing off the added chloroform recovered the initial diffusivity (Figure 2B,C). The application of 0.6 mM isoflurane, ∼2× of its EC_50_ value, similarly enhanced plasma-membrane *D* by ∼13% (Figure 2F,G).

We also examined the effects of anesthetics on the plasma-membrane diffusivity of cultured primary neurons. For flat two-dimensional regions like the soma (Figure 2H,I) and presumed growth cone (Figure S4), SM*d*M resolved *D* increases comparable to COS-7 cells. For one-dimensional neurites, whose nanoscale widths complicate diffusion analysis, we overcame the width confinement effect by fitting single-molecule displacements along the locally determined principal directions of diffusion,^28^ and thus also resolved similar anesthetic-induced *D* increases (Figure S6). We also noted slightly higher basal *D* values for the plasma membrane of cultured neurons, although this observation is complicated by intracellular and cell-to-cell heterogeneities and, thus, awaits more systematic examination. Together, SM*d*M has uncovered reversible *D* enhancements in both SLBs and live-cell plasma membranes upon anesthetic application.

### Anesthetic Insertion into Lipid Bilayers Leads to Area Expansion

The observed anesthetic-induced *D* enhancements imply changes in the lipid bilayer structure. Early results on the suppression of osmotic hemolysis in erythrocytes suggested that general anesthetics cause ∼0.4% cell-membrane area expansion at clinical doses.^44^

To visualize the likely expansion of the lipid bilayer, we prepared fluorescently labeled SLBs that partially covered the glass surface as islands and continuously recorded epifluorescence micrographs. Notably, the addition of 33 mM chloroform to the top PBS medium led to a rapid lateral expansion of the SLB islands (Figure 3A–C, Movie S1, and Figure S7). The expansion stabilized in ∼1 min, commensurate with the above SM*d*M-detected *D* rise time (Figure 1E), at which point ∼20% area expansion had occurred (Figure 3C, and Figure S7).

**Figure 3:**
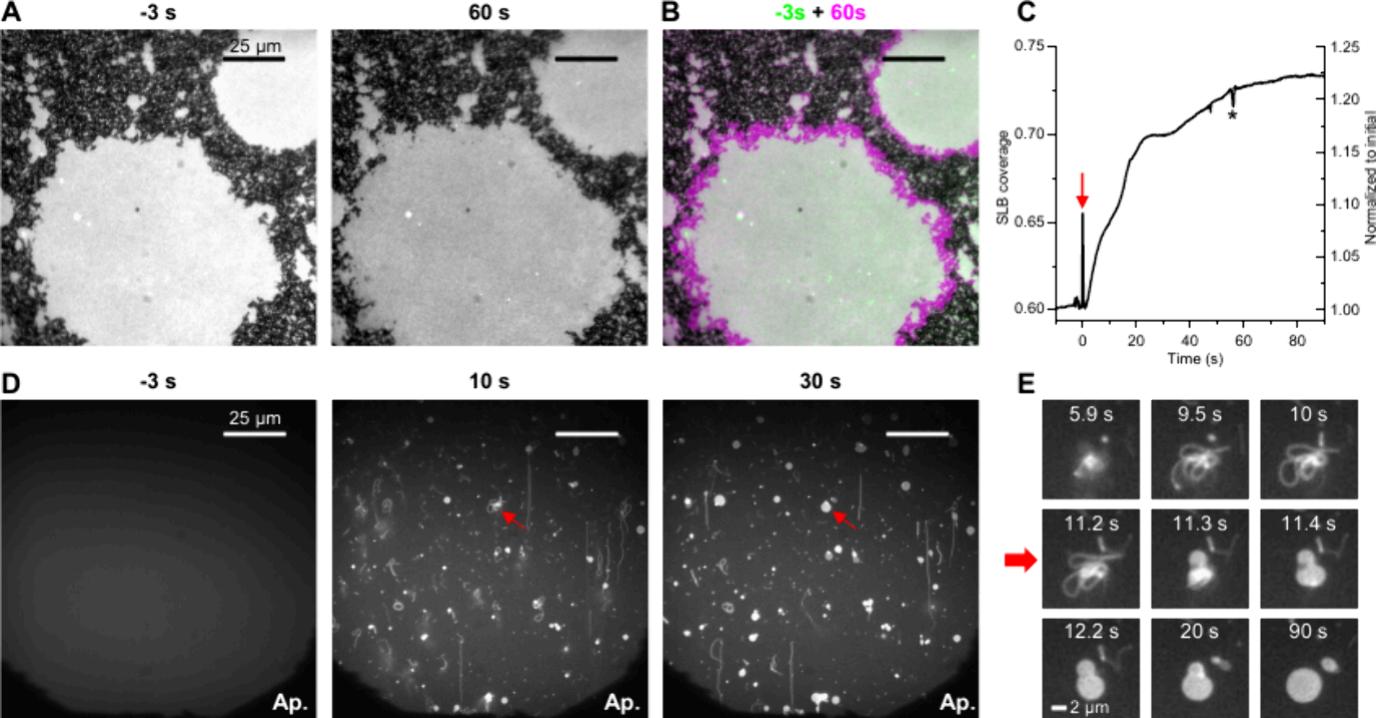
*In situ* fluorescence microscopy visualizes anesthetic-induced fast SLB area expansion and extrusion. (A) Fluorescently labeled DOPC SLBs partially covering the glass surface as bright islands, before (left) and 60 s after (right) adding 33 mM chloroform to the top PBS medium. (B) Overlaid images before (recolored green) and 60 s after (recolored magenta) chloroform addition, so consistently covered areas appear white whereas newly expanded areas appear magenta. (C) SLB surface coverage for the recorded field of view over time. Arrowhead: chloroform addition at 0 s. Asterisk: artifact due to adjustment of focus. (D) A fluorescently labeled DOPC SLB fully covering the glass surface, before (left), 10 s after (middle), and 30 s after (right) adding 33 mM chloroform to the top PBS medium. Ap.: Reduced aperture (microscope field diaphragm) to help focus on the featureless SLB. (E) Time series of the structure pointed to by the arrows in (D), highlighting fast structural changes.

For SLB fully covering the glass surface, we further made the intriguing observation that the addition of 33 mM chloroform led to the fast generation of >10 μm-long tubules (Figure 3D, Figure S8, and Movie S2). This result may be understood as chloroform-induced bilayer expansion leading to local extrusions out of the SLB surface at defect and/or nucleation sites, thus generating one-dimensional tubules that initially aligned in the flow direction of solution addition (Figure 3D) but then dynamically coalesced and stabilized into micrometer-sized domains (Figure 3E and Movie S2). Together, these results indicate that the partitioning of anesthetics into the lipid bilayer causes fast dilation and area expansion. Related SLB area expansion and tubule extrusion have been previously noted for the nonsteroidal anti-inflammatory drug ibuprofen at high dosages.^45^

### Anesthetic Application Increases Lipid-Bilayer Permeability to Chloride Ions

Our results above suggest that the insertion of anesthetics dilates and dilutes the lipid bilayer, thereby loosening lipid packing. Lateral diffusivity is a good indicator of lipid packing order: looser packing permits molecular motion and hence higher diffusivity.^38,46,47^ The lipid packing order also dictates bilayer permeability;^33,48^ for unilamellar vesicles of varied lipid compositions, higher membrane lateral diffusion coefficients correlate well with higher proton/hydroxide leak rates.^33^ Early studies have shown that general anesthetics increase the permeability of model lipid bilayers to potassium (K^+^) and other cations.^49–53^ However, due to the very low basal cation permeability levels, with which significant leaks take hours to achieve, these results are thought to indirectly reflect anesthetic-induced bilayer disordering rather than provide viable mechanisms for anesthesia.

We set out to interrogate whether anesthetic-induced lipid disordering may increase the bilayer permeability to the chloride anion (Cl^–^). Of major physiological ions, Cl^–^ has the highest lipid-bilayer permeability, >∼10^3^ over K^+^ and Na^+^.^54–56^ Moreover, as chloride’s equilibrium potential sits near the resting potential (∼−70 mV), increased chloride fluxes clamp the membrane potential and reduce neuronal excitability. Indeed, chloride channels like the γ-aminobutyric acid type A receptor (GABA_A_R) provide major neuronal inhibitory mechanisms and are sought-after targets of anesthesia mechanisms.^1–7^

To evaluate the chloride permeability of lipid bilayers, we employed a fluorescence quenching assay^56^ in which we encapsulated MQAE, a chloride-sensitive fluorescence dye, in a chloride-free buffer in 400 nm-sized large unilamellar vesicles (LUVs). After introducing these LUVs into a cuvette containing buffered potassium chloride (KCl), we monitored how fast the MQAE fluorescence was quenched as chloride gradually passed through the LUV membrane (Figure 4A).

**Figure 4:**
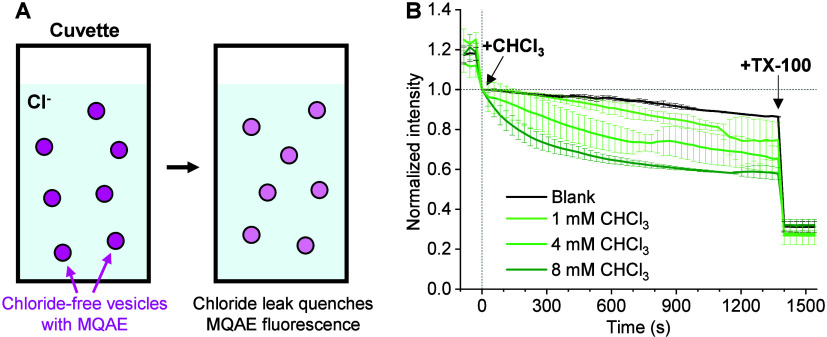
Liposome-based MQAE fluorescence quenching assay unveils increased lipid-membrane permeability to chloride (Cl^–^) in an anesthetic-concentration-dependent manner. (A) Schematic: 400 nm-sized LUVs are loaded with MQAE in a chloride-free buffer and then introduced into a cuvette containing buffered KCl for fluorescence measurement. Chloride leaking through the LUV membrane gradually quenches the MQAE fluorescence. (B) MQAE fluorescence intensity time traces with the addition of 0 (blank), 1, 4, and 8 mM chloroform to the cuvette at time 0, normalized to the intensity at time 0. Triton X-100 is applied at 1400 s in all runs to rupture the LUVs. Error bars: standard deviations between results from 3 runs under each condition.

For control samples in which a blank KCl buffer was added to the cuvette at time 0 to just dilute the LUVs, the MQAE fluorescence (black curve in Figure 4B) exhibited modest drops over 1400 s to 86% of the intensity at time 0 (*I*_0_), consistent with a moderate basal-level permeability of the lipid bilayer to chloride. The application of Triton X-100 to rupture the LUV and release MQAE into the KCl buffer next substantially quenched fluorescence to ∼30% of *I*_0_.

In comparison, adding a chloroform-containing KCl buffer to the cuvette at time 0 to yield a final chloroform concentration of 1 mM enhanced the MQAE fluorescence drop rate (light-green curve in Figure 4B), reaching ∼75% of *I*_0_ at 1400 s. Meanwhile, adding chloroform to 4 and 8 mM yielded progressively faster drops in MQAE fluorescence (green and dark-green curves in Figure 4B), down to 65% and 58% of *I*_0_ at 1400 s. Comparing the fluorescence intensities at an earlier time point of 600 s also showed contrasting signals of 96%, 91%, 76%, and 65% of *I*_0_ for 0, 1, 4, and 8 mM chloroform additions. Adding Triton X-100 at the end of the different runs yielded similar maximally quenched fluorescence at ∼30% of *I*_0_. In comparison, adding the nonanesthetic DMSO to 100 mM did not enhance the fluorescence drop rate over the blank addition (Figure S9A). As another control, adding 8 mM chloroform into samples with no chloride gradients across the LUVs did not induce a drop in MQAE fluorescence (Figure S9B). Our results indicate that the anesthetic loosening of the bilayer lipid packing substantially enhances chloride permeability.

## Conclusions

It has been a major challenge to elucidate how general anesthesia works given the immense structural diversity of anesthetics. Previous studies, mostly with suspended liposomes, have reported that general anesthetics lower the lipid packing order, increase bilayer fluidity, depress bilayer melting and critical temperatures,^10–16^ but minimally affect gramicidin dimerization in the bilayer.^40^

In this study, we began by examining the lateral diffusivity of both synthetic and cellular lipid membranes. Small changes in membrane diffusivity have been difficult to resolve: Traditional fluorescence microscopy approaches^38,57,58^ offer limited spatial resolution and precision, whereas single-molecule tracking^59,60^ is often limited by statistics, yielding scattered *D* values for individual tracks. Through the high-throughput sampling of >10^5^ single-molecule displacements, SM*d*M attains ∼0.3% *D* precision at 95% CI, and so comfortably resolved the subtle *D* enhancements induced by anesthetics. The super-resolution mapping capabilities of SM*d*M further uniquely helped resolve cellular patterns to elucidate plasma-membrane diffusivity. For SLBs, SM*d*M thus resolved a converging ∼5% increase in *D* for diverse anesthetics at their contrasting EC_50_ values, and showed that the *D* enhancement scaled near linearly with the anesthetic dose. Through *in situ* SM*d*M, we further found that the anesthetic enhancement of bilayer diffusivity was both fast-acting and quickly reversed. For both cultured fibroblasts and neurons, SM*d*M super-resolution *D* maps overcame spatial inhomogeneities to resolve ∼10% increases in the diffusivity of plasma membranes for anesthetics applied at nominal concentrations 1–3× of their EC_50_ values, thus demonstrating that anesthetics’ act on lipid bilayers gives rise to substantial, detectable changes in the physical properties of the plasma membrane.

With *in situ* fluorescence microscopy, we next unveiled anesthetic-induced fast area expansion and extrusion of SLBs. Together with the SM*d*M data above, these results suggest that anesthetic insertion dilates the lipid bilayer, thus, loosening lipid packing to accommodate molecular diffusion. Employing a liposome-based fluorescence quenching assay, we next showed that anesthetics substantially enhanced bilayer permeability to chloride in a concentration-dependent fashion. Chloride has orders-of-magnitude higher bilayer permeability over other physiological ions, and chloride fluxes provide a major path to suppress neuronal excitability. Whereas ample past work has proposed that anesthetics act by activating chloride channels,^1–7^ blocking or knocking out chloride channels does not substantially reduce the potency of volatile anesthetics in animals.^5^ Our discovery of anesthetic-enhanced lipid-bilayer permeability to chloride, awaiting future comparison and validation with electrophysiological studies in cells, offers complementary possibilities. Early studies showed that high pressures antagonize anesthesia in animals.^61–63^ Conversely, deep-sea animals adapted to high pressures exhibit pressure-reversible paralysis upon depressurization.^64^ These observations may be explained by considering that high pressures compact bilayer lipid packing^16,65–67^ and thus reduce chloride permeability.

Together, while highlighting the outstanding fidelity and nanoscale mapping capabilities of SM*d*M in quantifying molecular diffusivity, our results shed light on the long-standing puzzle of the molecular mechanism of general anesthesia. Extending the strategies developed in this work to other drug-membrane interactions presents exciting opportunities.

## Materials and Methods

### Supported Lipid Bilayers

Fluorescently labeled SLBs were prepared following typical protocols.^31^ To prepare SLBs for SM*d*M, chloroform stock solutions were prepared from the following lipids: DOPC (Avanti Polar Lipids 850375C), brain sphingomyelin (Avanti Polar Lipids 860062), cholesterol (Sigma C8667), and Texas Red-DHPE (Invitrogen T1395MP). 25 mL round-bottom flasks were cleaned and etched by a heated 3:1 H_2_SO_4_:H_2_O_2_ (30%) mixture, rinsed with Milli-Q water, dried with N_2_, and baked dry. The above lipid stock solutions were mixed at the desired ratios and added to the round-bottom flask for a total mass of 1 mg lipid. The solvent was removed under a stream of nitrogen gas and placed under vacuum for >30 min. The resulting lipid film was rehydrated in 60 °C Milli-Q water and vortexed to a final concentration of 1 mg/mL multilamellar vesicle (MLV) solution. The MLV solution was then tip sonicated (VWR, 76193-590) at 40% power using a two-second on, one-second off sequence for 3–4 min to create ∼100 nm small unilamellar vesicles (SUVs). Then 25 mm #1.5 glass coverslips were cleaned and etched with a heated 3:1 H_2_SO_4_:H_2_O_2_ (30%) mixture, rinsed with Milli-Q water, dried with N_2_ gas, and then mounted with Attofluor cell chambers (Invitrogen A7816). The SUV solution was diluted as a 5:4:1 mixture of PBS (phosphate-buffered saline):H_2_O:SUV solution and deposited on the etched coverslips. Excess vesicles were removed after 20 min by washing with 3 mL of Milli-Q water followed by >3 mL PBS for a final volume of 1 mL. To prepare SLBs for epifluorescence imaging, TopFluor PC (Avanti Polar Lipids 810281) was added to DOPC at 0.1%. Fully covered SLBs were similarly prepared as above. To form patchy SLBs that covered the glass surface as islands, the 1 mg/mL MLV solution was not sonicated but was diluted as a 2:1:1 mixture of PBS:H_2_O:MLV solution for a 1 mL final volume. This solution was added to the etched glass coverslips and incubated for 20 min, and then washed with 3 mL of Milli-Q water followed by >3 mL of PBS. Epifluorescence imaging of SLBs was performed on an Olympus IX73 inverted microscope with a water-immersion objective (Olympus, UPLSAPO60XW, NA 1.2) under low illumination. Wide-field images were continuously recorded using an sCMOS camera (Zyla 4.2, Andor) at 10 fps.

### Cell Culture

COS-7 cells (UC Berkeley Cell Culture Facility) were cultured in Dulbecco’s Modified Eagle’s Medium (DMEM, high glucose) in 5% CO_2_ at 37 °C. For SM*d*M, cells were plated on 25 mm glass coverslips and imaged in phenol-red-free Leibovitz’s L-15 medium (ThermoFisher, 21083027) and 20 mM HEPES (pH = 7.3; Thermo Fisher 15630080) with the addition of 0.5–2 nM BDP–TMR–alkyne (Lumiprobe A24B0).^28^ Primary rat hippocampal neurons were purchased from BrainBits and cultured in NbActiv4 medium (BrainBits NB4). They were cultured on 25 mm coverslips or 8-well chambered coverslips (Nunc Lab-Tek II) coated with poly-d-lysine per recommended protocols for 1–2 weeks and then imaged in phenol-red-free Hibernate A medium (BrainBits, HAPR) with the addition of BDP–TMR–alkyne.

### Application of Anesthetics

The following anesthetics and nonanesthetics were used: chloroform (Fisher, C606-4), halothane (Sigma, B4388), isoflurane (Fisher Scientific, L17315.06), diethyl ether (Sigma, 296082), and 1,2-dichlorohexafluorocyclobutane (F6) (Sigma, S643459). Saturated solutions were first prepared for each anesthetic by mixing an excess amount with PBS (for SLBs), L-15 (for COS-7 cells), or Hibernate A (for neurons) media, vortexing, and allowing the solution to settle overnight. For each desired final concentration, a 2× solution was first prepared from the saturated solution, and this solution was used to replace 50% of the medium on top of the SLB or cell sample. A 25 mm glass coverslip was immediately placed flat over the 20 mm opening of the Attofluor cell chamber to create a seal and reduce anesthetic evaporation during the experiments.

### SMdM

SM*d*M was performed as described previously^27–29^ on a Nikon Ti-E inverted fluorescence microscope at room temperature (22 ± 1 °C). Briefly, a 561-nm laser was focused at the back focal plane of an oil-immersion objective lens (CFI Plan Apochromat Lambda 100×, NA = 1.45). A motorized stage shifted the laser beam toward the edge of the objective lens, allowing the laser to enter the sample at the critical angle of the glass-sample interface to achieve total internal reflection (TIR) illumination for the SLB samples or slightly below the critical angle to illuminate ∼1 μm into the cell samples. The laser illumination angle remained unchanged in the experiment, and we expect the added ∼1 mM anesthetics to minimally alter the refractive index of the medium. Wide-field single-molecule images were recorded with an EM-CCD instrument (Andor iXon Ultra 897) that operated continuously at 109.3 frames per second. The laser was modulated to excite at the center of every frame as 2.5 ms-duration pulses at a fixed time separation of Δ*t* = 9.15 ms. The low illumination duty cycle allowed fresh fluorescent probes to diffuse into the imaging area from outside the illuminated region, thus maintaining a suitable density of single molecules in the field of view.^29^ Typical SLB runs executed ∼2 × 10^4^ frames in ∼3 min. 10^5^–10^6^ single-molecule displacements *d* were extracted between all consecutive frames^28^ for fitting to the probability model below (eq 1) to obtain a *D* value. For the *in situ* monitoring of *D* changes during chloroform addition and removal, SM*d*M ran continuously for ∼2 × 10^5^ frames over ∼30 min, and the accumulated single-molecule displacements were segmented for every 1093 frames for fitting to the probability model below (eq 1) to obtain a *D* value for the 10 s duration. Typical runs for cell samples executed ∼5 × 10^4^ frames in ∼8 min, and the accumulated single-molecule displacements were spatially binned onto a 120 nm × 120 nm grid, so that displacements that fell into each bin were fit to the probability model below (eq 1) to obtain a local *D* value to achieve super-resolution mapping. The fitting probability model for displacement *r* is based on normal diffusion:

1 $$P\left(r\right)=\frac{2r}{a}\;\exp\left(-\frac{r^{2}}{a}\right)+br$$

Here *a* = 4*D*Δ*t*, with *D* being the diffusion coefficient and Δ*t* = 9.15 ms being the above fixed time separation. *b* accounts for a uniform background.^27,29^ Fitting was done through maximum likelihood estimation using MATLAB, which returned 95% confidence intervals for *D*. For spatially binned data, the fitted *D* value in each bin was assigned a color on the continuous *D* scale for rendering into a color-coded super-resolution *D* map.

### Liposome Fluorescence Quenching Assay

A liposome-based MQAE fluorescence quenching assay^56^ was employed to examine the chloride permeability of lipid bilayers. DOPC was deposited at the bottom of an acid-etched and dried round-bottom flask, as above. The lipid film was rehydrated in 0.5 mL of a buffer consisting of 200 mM potassium gluconate (Thermo Fisher B25135.18), 20 mM HEPES (pH = 7.3; Thermo Fisher 15630080), and 10 mM MQAE (Enzo Life Science ENZ-52156) to a final lipid concentration of 20 mg/mL. The suspension was then extruded through 400 nm polycarbonate membrane filters (Whatman 10417104) using a mini-extruder (Avanti Polar Lipids, 610000) to form dye-encapsulated large unilamellar vesicles (LUVs). Unencapsulated dye was removed by filtration twice through desalting NAP-5 Sephadex G-25 columns (Cytiva, 17085302) using a buffer containing 210 mM potassium gluconate and 20 mM HEPES. For the MQAE fluorescence quenching assay, the MQAE-encapsulated vesicle solution was mixed at a 1:7 ratio in a quartz cuvette with a buffer containing 210 mM KCl (Sigma, P9541), 20 mM HEPES, and 3.5 μM valinomycin (Sigma V0627). MQAE emission was repeatedly collected every 28 s on a fluorescence spectrometer (Horiba Duetta) under 354 nm excitation. After 2.5 min, a blank buffer of 210 mM KCl and 20 mM HEPES, or the same buffer with the addition of 8, 32, or 64 mM chloroform, was added to the cuvette at a 1:7 volume ratio to yield final chloroform concentrations of 0, 1, 4, and 8 mM. This chloroform/blank-addition step diluted the starting MQAE LUV concentration by 1/7; we define the time point of this step as time 0 and the fluorescence intensity at this time point as the starting intensity *I*_0_ when plotting the MQAE fluorescence intensity time traces. At 1400 s, a 10 mM solution of Triton X-100 was added to the cuvette at a 1:8 volume ratio to rupture all the vesicles and release MQAE into the KCl buffer.

## Supplementary Material







## Notes

Emission spectra of Texas Red-DHPE in PBS-suspended DOPC SUVs with varied chloroform added; SM*d*M results on DOPC SLBs with the addition of isoflurane, DMSO, and F6; SM*d*M results on SLBs formed with a saturated/unsaturated lipid mixture; additional SMLM and SM*d*M images for anesthetics applied to live cells; *in situ* visualization of chloroform-induced diffusivity changes in the plasma membrane; principal-direction SM*d*M super-resolution mapping of membrane diffusivity in neurites; additional analysis for the *in situ* fluorescence microscopy of chloroform-induced SLB expansion and tubule extrusion; and additional control data for the liposome-based MQAE quenching assay (PDF)
Time series for the *in situ* fluorescence microscopy of chloroform-induced SLB expansion (MP4)
Time series for the *in situ* fluorescence microscopy of chloroform-induced SLB tubule extrusion (MP4)
