# Supplementary material for: Single-Molecule and Super-Resolution Diffusion Quantification Unveils Reversible Enhancement of Lipid-Membrane Diffusivity by General Anesthetics
Source: ACS Nano. 2025 Nov 13;19(46):39864–72. doi: 10.1021/acsnano.5c13446 (PMC12659419; doi:10.1021/acsnano.5c13446)
Supplement: Supplementary file 1 [file nn5c13446_si_001.pdf]

Supporting Information

Single-molecule and super-resolution diffusion quantification unveils reversible enhancement of lipid-membrane diffusivity by general anesthetics

Tyler Jepson, Hansen Jin, Chun Ying Wu, Wan Li, Ke Xu*

Department of Chemistry, University of California, Berkeley, California 94720, United States

California Institute for Quantitative Biosciences, University of California, Berkeley, CA 94720, United States

* Corresponding author: xuk@berkeley.edu (K.X.)

Supplementary Figures

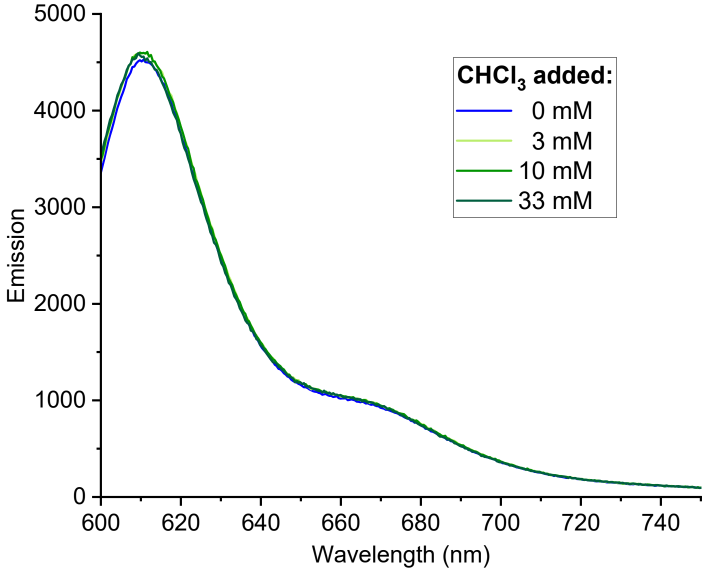

Figure S1. The application of chloroform does not noticeably alter the fluorescence emission of the dye-labeled lipid. Here, DOPC and Texas Red-DHPE were mixed at a molar ratio of 99.9% and 0.1%, with which PBS-suspended SUVs were prepared. The suspended SUVs were then mixed with PBS containing varying amounts of chloroform to achieve the same final lipid concentration of 0.125 mg/mL and 0, 3, 10, and 33 mM final concentrations of chloroform. Fluorescence emission was recorded using a Duetta spectrometer (HORIBA Instruments) under 594-nm excitation. Shown spectra are the average of 6 runs for the no-chloroform condition and the averages of 3 runs for the other conditions.

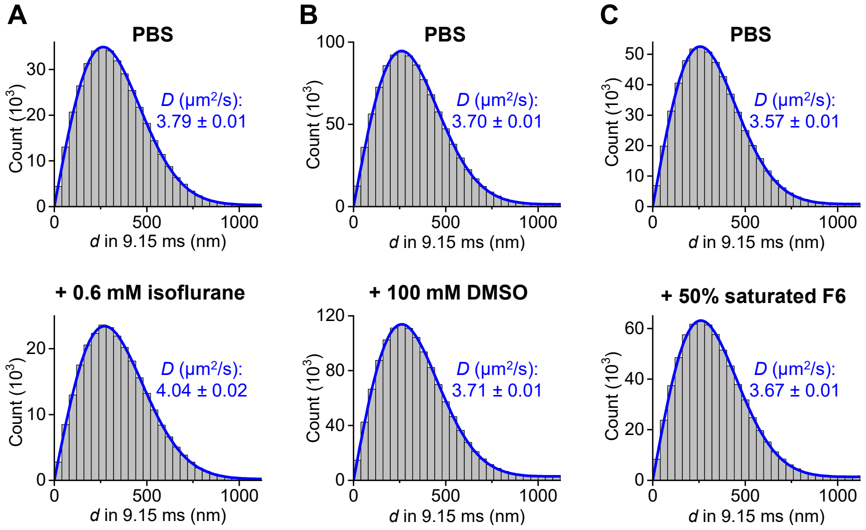

Figure S2. SM*d*M measurement of DOPC SLB diffusivity with the addition of isoflurane, DMSO, and F6. Histograms: distribution of SM*d*M-recorded single-molecule displacement *d* of Texas Red-DHPE in the SLB, at a fixed time separation of Δt = 9.15 ms. Blue curves: Fits to a normal diffusion model, with resultant *D* and 95% CI marked in each graph. (**A**) Results without (**top**) and with (**bottom**) the addition of 0.6 mM isoflurane to the top PBS medium. (**B**) Results without (**top**) and with (**bottom**) the addition of 100 mM DMSO to the top PBS medium. (**C**) Results without (**top**) and with (**bottom**) the addition of an equal volume of F6-saturated PBS to the top PBS medium. The water solubility of F6 is ~225 μM,[1,2] so F6 in the 50%-saturated solution is ~112 μM. The presumed EC_{50} (no actual anesthesia) of F6 is ~16 μM.[3] The very low water solubility of F6 may be a confounding factor in its effective delivery. Experiments were repeated 3 times for each condition, with similar results observed.

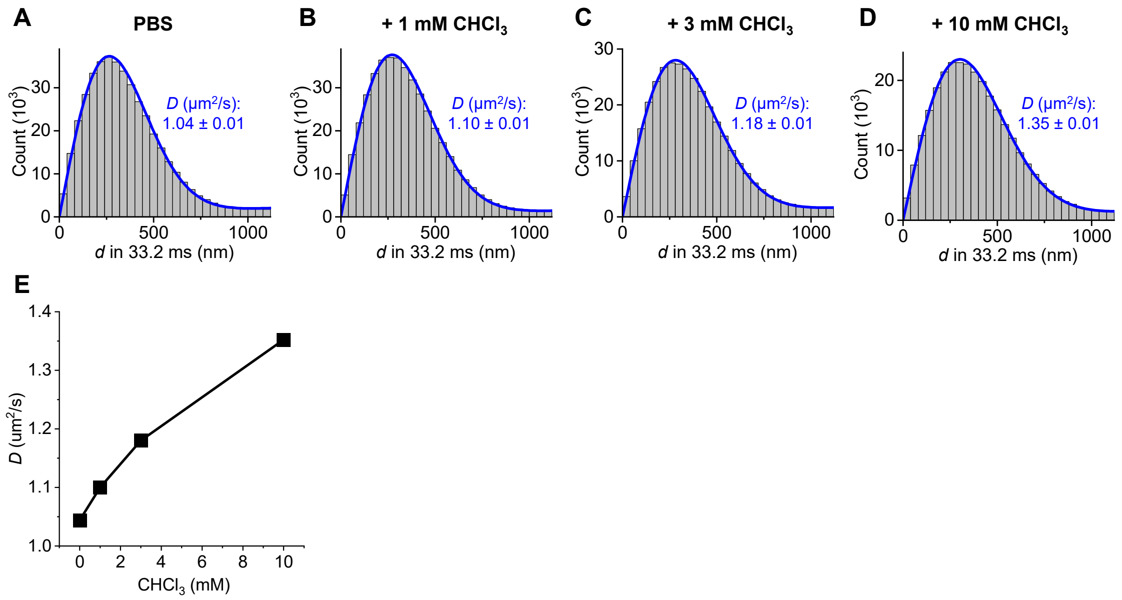

Figure S3. SM*d*M results on SLBs formed with a saturated/unsaturated lipid mixture of DOPC, brain sphingomyelin, and cholesterol at a 1:1:1 mole ratio. (**A**) Histogram: distribution of SM*d*M-recorded single-molecule displacement *d* of Texas Red-DHPE in the mixed-lipid SLB in PBS, at a fixed time separation of Δt = 33.2 ms. Here a reduced camera framerate of 30.2 fps was used to quantify the slower diffusion rates. Blue curve: Fit to a normal diffusion model, yielding D = 1.04 ± 0.01 $\mu m^2/s$ (95% CI). (**B-D**) Distributions of single-molecule displacements for the same SLB sample after including 1, 3, and 10 mM chloroform in the top PBS medium. Blue curves: Fits to a normal diffusion model, yielding D = 1.10 ± 0.01, 1.18 ± 0.01, and 1.35 ± 0.01 $\mu m^2/s$ (95% CI). (**E**) SM*d*M-determined D values as a function of chloroform concentration in the top PBS medium.

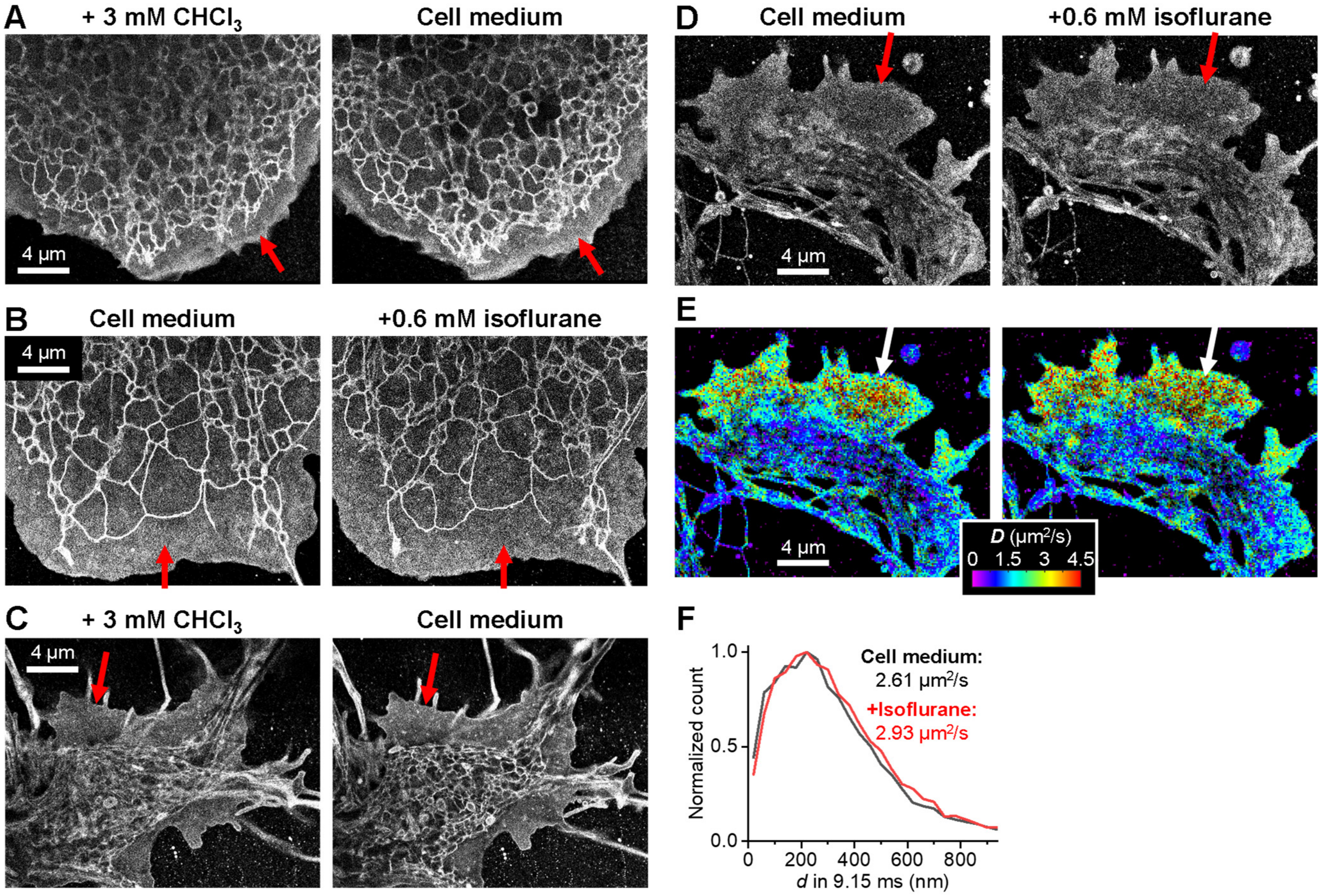

Figure S4. Additional SMLM and SM*d*M images for anesthetics applied to live cells. (**A**) SMLM images of a COS-7 cell in the presence of 3 mM chloroform (**left**) and after washing off with medium (**right**), constructed from the single-molecule localizations of the SM*d*M data shown in Fig. 2D. Arrows point to plasma-membrane regions from which distributions of single-molecule displacements are plotted in Fig. 2E. (**B**) SMLM images of a COS-7 cell before (**left**) and after (**right**) adding 0.6 mM isoflurane to the cell medium, constructed from the single-molecule localizations of the SM*d*M data shown in Fig. 2F. (**C**) SMLM images of the soma of a cultured neuron in the presence of 3 mM chloroform (**left**) and after washing off with medium (**right**), constructed from the single-molecule localizations of the SM*d*M data shown in Fig. 2H. (**D,E**) SMLM images (**D**) and color-coded SM*d*M diffusivity maps (**E**) of BDP-TMR-alkyne for the presumed growth cone of a cultured neuron before (**left**) and after (**right**) adding 0.6 mM isoflurane to the cell medium. (**F**) Distributions of single-molecule displacements for the lamellipodia region of the plasma membrane, as pointed to by arrows in (D,E), to which D values are fitted and marked in the graph.

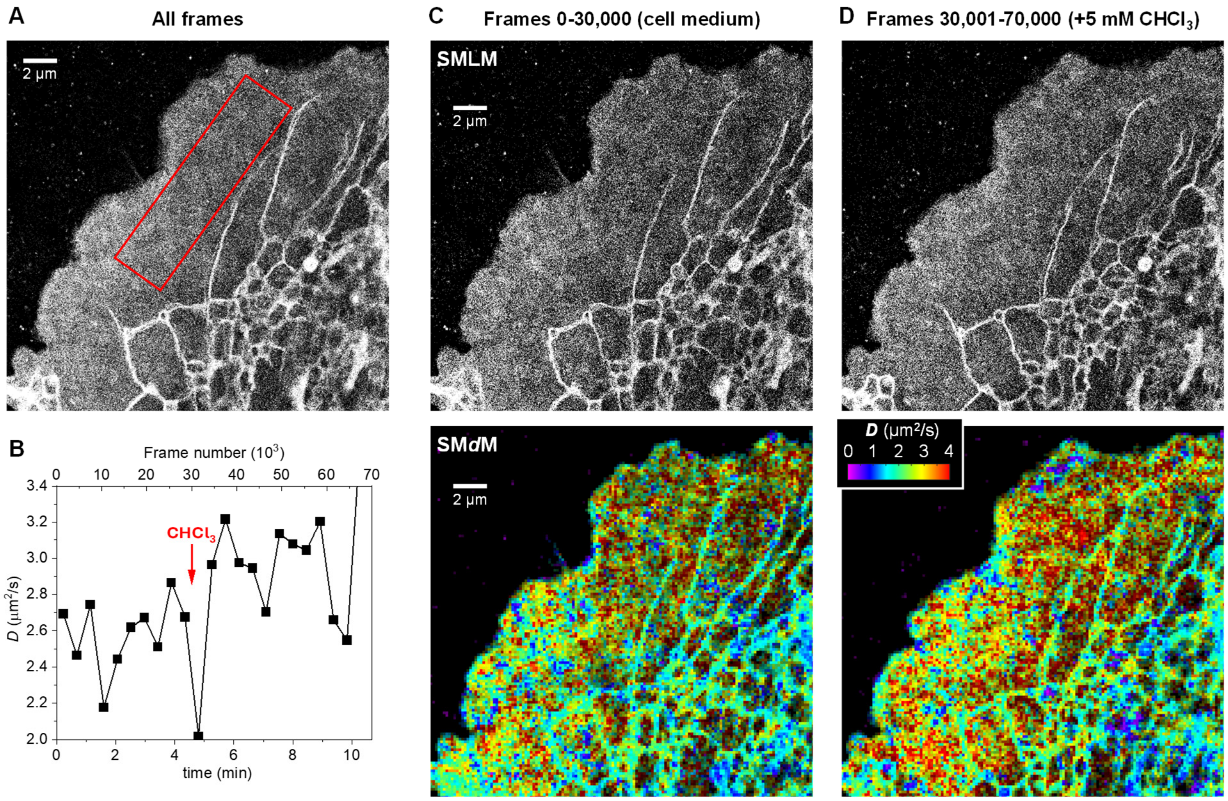

Figure S5. *In situ* visualization of chloroform-induced diffusivity changes in the plasma membrane. (**A**) SMLM image constructed from the localized BDP-TMR-alkyne molecules in an SM*d*M dataset in which 5 mM chloroform was added to the medium at Frame 30,000. (**B**) SM*d*M-determined *D* values versus time (bottom axis) and frame number (top axis) for the boxed region in (A). Each data point is from fitting 3,000 consecutive frames (27.4 s) of SM*d*M data. (**C**) SMLM image (**Top**) and SM*d*M diffusivity map (**Bottom**) constructed from the first 30,000 frames before chloroform addition. (**D**) SMLM image (**Top**) and SM*d*M diffusivity map (**Bottom**) constructed from Frames 30,001-70,000 after chloroform addition.

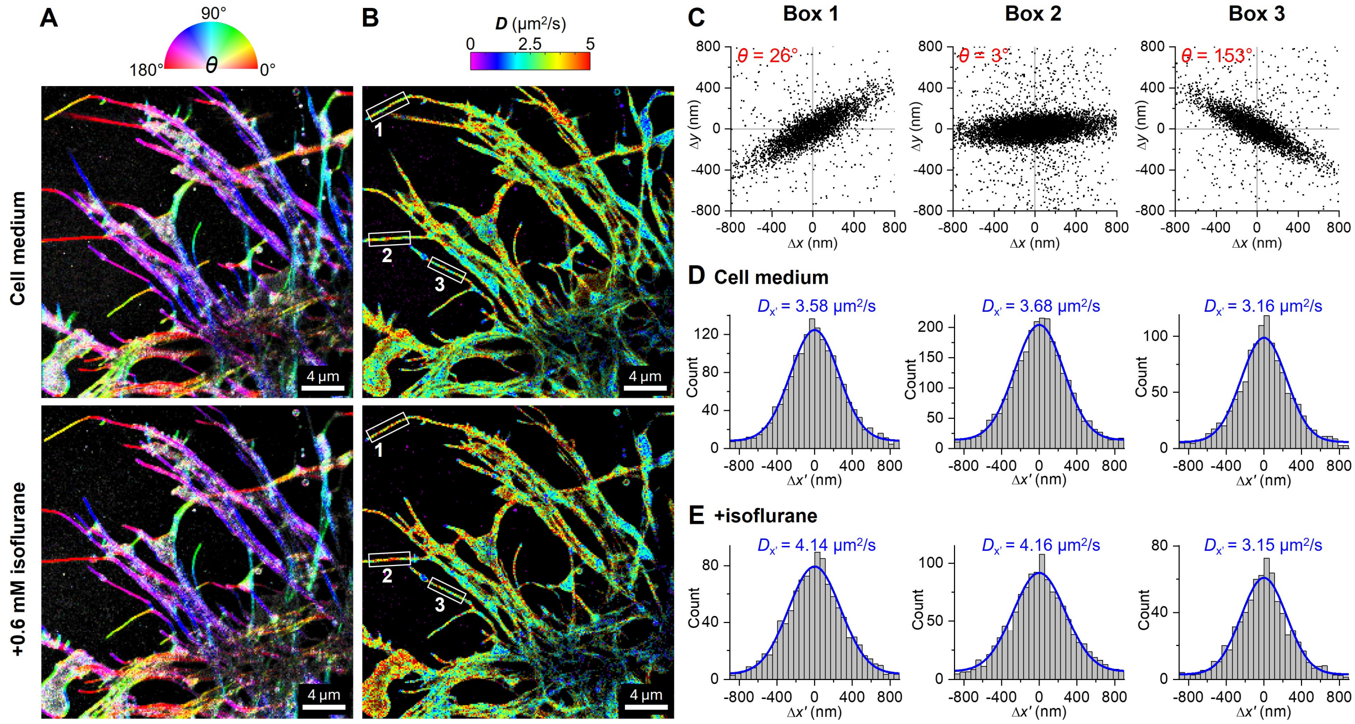

Figure S6. Principal-direction SM*d*M (pSM*d*M) super-resolution mapping of membrane diffusivity in neurites. Here, due to the nanoscale diameters of the neurites, diffusion is unrestrained along the neurites but strongly confined in the width direction. This same issue is analyzed in detail in our previous work on membrane diffusion in endoplasmic-reticulum tubules, for which we developed pSM*d*M to recover meaningful diffusion rates.[4,5] In pSM*d*M, for each spatial bin (120 nm × 120 nm in this study), we analyze its accumulated two-dimensional single-molecule displacement vectors to first determine its local principal direction of diffusion θ. The single-molecule displacement vectors are then projected along local θ for fitting to a one-dimensional diffusion model to extract the local diffusion coefficient D. (**A**) Color maps presenting the calculated local θ for BDP-TMR-alkyne in the neurite membranes of cultured neurons, before (**top**) and after (**bottom**) applying 0.6 mM isoflurane. (**B**) Corresponding pSM*d*M D maps based on one-dimensional diffusion fits along local θ. (**C**) Two-dimensional plots of single-molecule displacement vectors (Δt = 9.15 ms), for the three boxed neurites marked in (B) before isoflurane application. Principal directions θ are calculated as 26°, 3°, and 153° for the three regions, respectively. (**D**) Histograms: Distributions of the displacements in (C) after projected along their respective θ directions. Blue curves: Fits to a one-dimensional diffusion model, with resultant D values marked in each plot. (**E**) Similar to (D), but for the same regions after the application of 0.6 mM isoflurane. Increased D is found for Boxes 1 and 2 but not for Box 3. Although we do not understand the observed spatial heterogeneities, a general increase in D is visualized by the overall redshift of the pSM*d*M color map in (B).

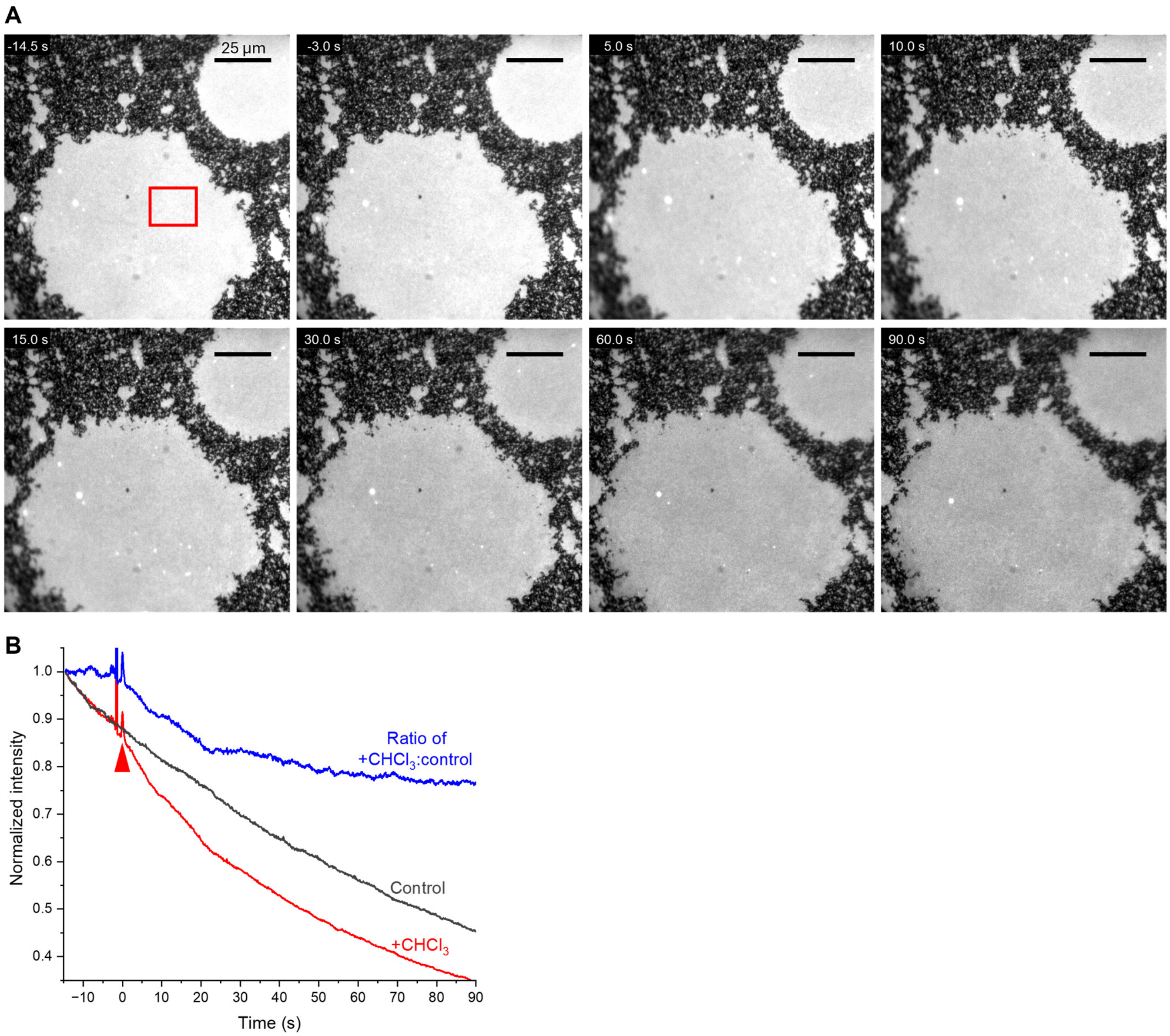

Figure S7. Additional analysis for the *in situ* fluorescence microscopy of chloroform-induced SLB expansion. (**A**) Additional time series for the fluorescence images shown in Fig. 3AB and Movie S1. Fluorescently labeled DOPC SLBs initially cover the glass surface as bright islands. At time 0, an equal volume of PBS containing 66 mM chloroform is added to the original top PBS medium to achieve a final chloroform concentration of 33 mM. (**B**) Gray curve: photobleach decay of the fluorescence signal of a control SLB sample in PBS. This decay trend is used to normalize the fluorescence image intensity shown in (A). Red curve: fluorescence signal for the boxed region in (A). Arrowhead: disturbance due to chloroform addition at 0 s. Blue curve: ratio of the above two, showing that the addition of chloroform induced an additional ~20% drop in the fluorescence signal for the boxed region due to a commensurate expansion of the SLB area (Fig. 3C) and hence dilution of the fluorescently labeled lipid.

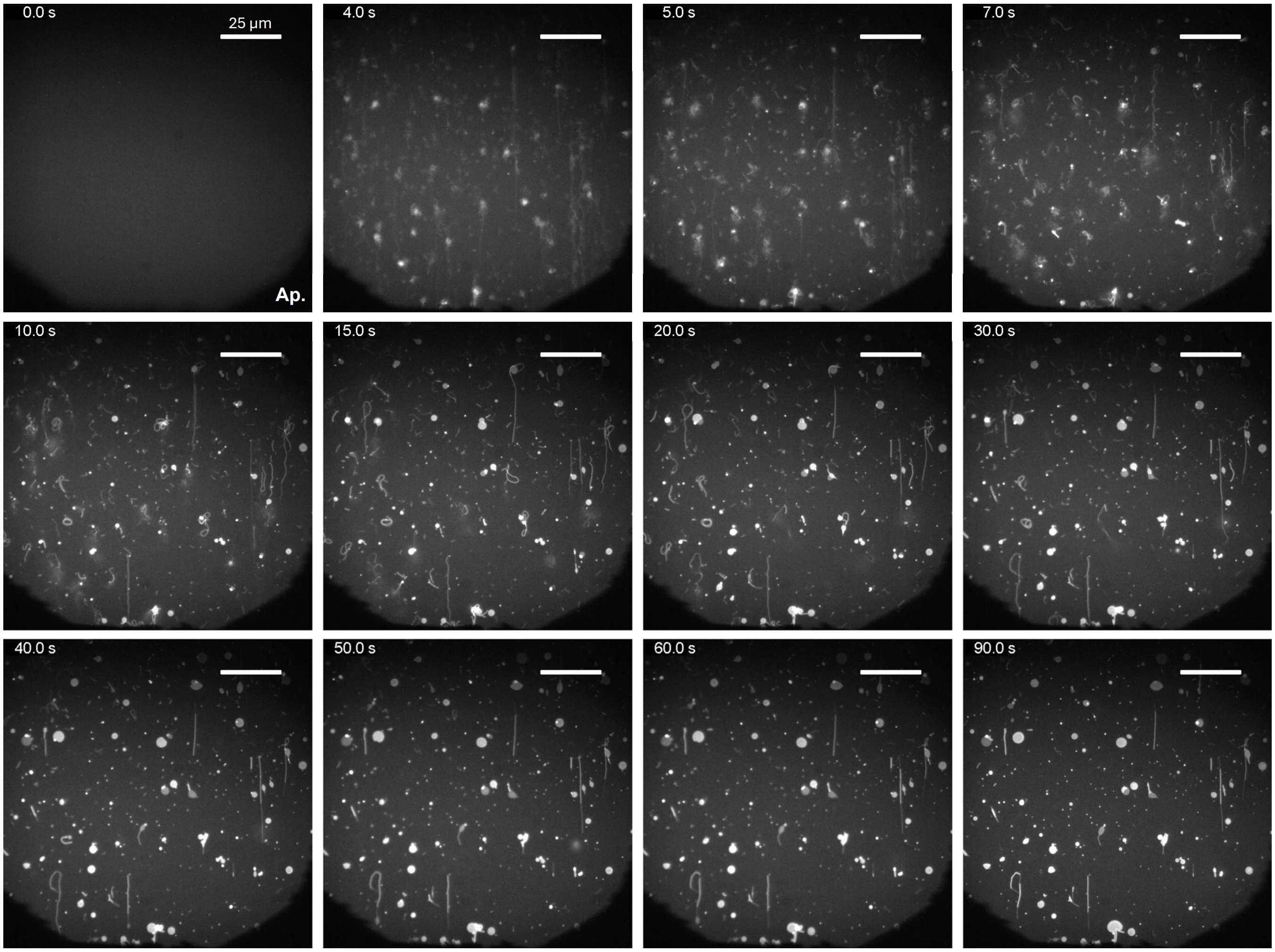

Figure S8. Additional time series of *in situ* fluorescence microscopy images of chloroform-induced tubule extrusion for a fluorescently labeled SLB fully covering the glass surface, as shown in Fig. 3D-E and Movie S2. At time 0, an equal volume of PBS containing 66 mM chloroform is added to the original top PBS medium to achieve a final chloroform concentration of 33 mM. This operation generated a flow that aligned the emerging tubules.

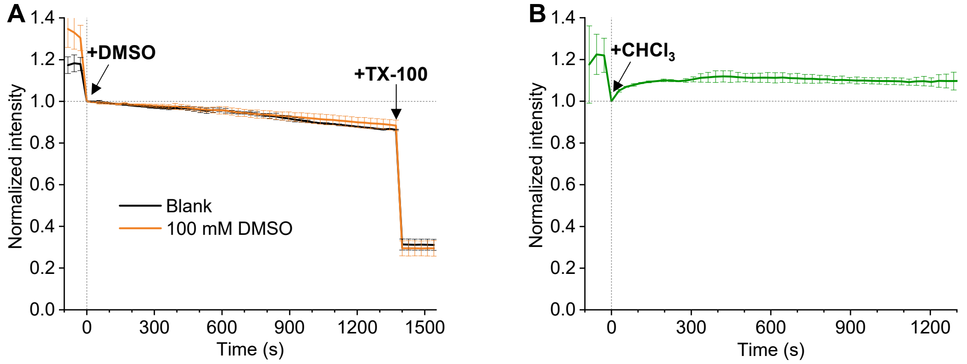

Figure S9. (**A**) Liposome-based MQAE fluorescence quenching assay as described in Fig. 4 but for DMSO. 100 mM DMSO is added to the cuvette at time 0, and MQAE fluorescence intensity is normalized to the intensity at time 0. Triton X-100 is applied at 1,400 s to rupture the LUVs. Error bars: standard deviations between results from 3 runs. (**B**) Control experiment: MQAE-loaded LUVs were added to a cuvette containing a chloride-free buffer of 20 mM HEPES and 210 mM potassium gluconate, and 8 mM chloroform was added at time 0. Error bars: standard deviations between results from 2 runs.

Captions to Movies

Movie S1. Time series for the *in situ* fluorescence microscopy of chloroform-induced SLB expansion. Fluorescently labeled DOPC SLBs initially cover the glass surface as bright islands. At time 0, an equal volume of PBS containing 66 mM chloroform is added to the original top PBS medium to achieve a final chloroform concentration of 33 mM. Scale bar: 25 μm.

Movie S2. Time series for the *in situ* fluorescence microscopy of chloroform-induced tubule extrusion for a fluorescently labeled SLB fully covering the glass surface. At time 0, an equal volume of PBS containing 66 mM chloroform is added to the original top PBS medium to achieve a final chloroform concentration of 33 mM. This operation generated a flow that aligned the emerging tubules. Scale bar: 25 μm.

References for Supplement

(1) Raines, D. E.; Claycomb, R. J.; Forman, S. A. Nonhalogenated Anesthetic Alkanes and Perhalogenated Nonimmobilizing Alkanes Inhibit $A_4\beta_2$ Neuronal Nicotinic Acetylcholine Receptors. *Anesth. Analg.* **2002**, *95*, 573-577.

(2) Recio-Pinto, E.; Montoya-Gacharna, J. V.; Xu, F.; Blanck, T. J. J. Isoflurane, but Not the Nonimmobilizers F6 and F8, Inhibits Rat Spinal Cord Motor Neuron Ca_v1 Calcium Currents. *Anesth. Analg.* **2016**, *122*, 730-737.

(3) Perouansky, M. Non-Immobilizing Inhalational Anesthetic-Like Compounds. In *Modern Anesthetics*; Schüttler, J., Schwilden, H., Eds.; Springer Berlin Heidelberg: Berlin, Heidelberg, 2008; pp 209-223.

(4) Yan, R.; Chen, K.; Xu, K. Probing Nanoscale Diffusional Heterogeneities in Cellular Membranes through Multidimensional Single-Molecule and Super-Resolution Microscopy. *J. Am. Chem. Soc.* **2020**, *142*, 18866-18873.

(5) Li, W.; Xu, K. Super-Resolution Mapping and Quantification of Molecular Diffusion Via Single-Molecule Displacement/Diffusivity Mapping (SM*d*M). *Acc. Chem. Res.* **2025**, *58*, 1224-1235.
